# Clinical significance of plasma cell-free DNA mutations in *PIK3CA*, *AKT1*, and *ESR1* gene according to treatment lines in ER-positive breast cancer

**DOI:** 10.1186/s12943-018-0808-y

**Published:** 2018-02-26

**Authors:** Takashi Takeshita, Yutaka Yamamoto, Mutsuko Yamamoto-Ibusuki, Mai Tomiguchi, Aiko Sueta, Keiichi Murakami, Hirotaka Iwase

**Affiliations:** 1 Department of Breast and Endocrine surgery, Graduate School of Medical Science, Kumamoto University, 1-1-1 Honjo, Chuo-ku, Kumamoto, 860-8556 Japan; 2 Department of Molecular-Targeting Therapy for Breast Cancer, Kumamoto University Hospital, 1-1-1 Honjo, Chuo-ku, Kumamoto, 860-8556 Japan

**Keywords:** Estrogen receptor-positive breast cancer, Cell-free DNA, *PIK3CA* mutations, *AKT1* mutation, *ESR1* mutations

## Abstract

The somatic activation of PI3K/AKT pathway mutations, *PIK3CA* and *AKT1,* and *ESR1* mutations in plasma cell-free DNA (cfDNA) has been studied as a non-invasive procedure to quickly assess and monitor disease progression or therapeutic effect in breast cancer (BC) patients, but the clinical significance of these mutations in late treatment lines (TLs) remains unclear. The subjects of this study were a total of 251 plasma samples from 128 estrogen receptor-positive (ER+) BC patients. Of these plasma samples, 133 were from 73 primary BC (PBC) patients, and 118 plasma samples were from 68 metastatic BC (MBC) patients. We developed droplet digital PCR (ddPCR) assays to verify the clinical significance of *PIK3CA*, *AKT1*, and *ESR1* mutations in these patients. cfDNA *PIK3CA* mutations were observed in 15.1% of the PBC patients, while a cfDNA *AKT1* mutation was observed in 1.4% of patients, and cfDNA *ESR1* mutations were observed in 2.7% of patients. Patients with detectable cfDNA *PIK3CA* mutations were not associated with clinical outcomes. According to the TL, the prevalence of the *PIK3CA* and *ESR1* mutations in cfDNA were lower in early TLs compared with late TLs. In the early TL group, patients with cfDNA *PIK3CA* mutations had a shorter time to treatment failure (TTF) than patients without mutations (*P* = 0.035). However, there was no statistically significant difference between patients with or without cfDNA *ESR1* mutations. However, in the late TL group, patients with cfDNA *ESR1* mutations had a shorter TTF than patients without mutations (*P* = 0.048). However, there was no statistically significant difference between patients with or without cfDNA *PIK3CA* mutations. Since the prevalence of cfDNA *AKT1* mutation is low in both PBC and MBC patients, the impact of *AKT1* mutations on the prognosis remains unclear. We have demonstrated the difference in the clinical significance of the hotspot *PIK3CA, AKT1,* and *ESR1* mutations in cfDNA for each TL in ER+ BC patients.

**Electronic supplementary material:**

The online version of this article (10.1186/s12943-018-0808-y) contains supplementary material, which is available to authorized users.

Endocrine therapy (ET) resistance occasionally occurs during the treatment of primary breast cancer (PBC) and inevitably results in metastatic BC (MBC). Recently, the focused mechanisms of ET resistance include hyperactivation of *PI3K/AKT* pathway. Importantly, somatic activating mutations of *PIK3CA* and *AKT1* affect the magnitude of *PI3K/AKT* activation [1]. Meta-analysis of the BC literature shows that the *PIK3CA* mutation is present in 20%-40% of all BCs, making this gene the second most frequently mutated in BC, with most mutations being expressed in ‘hotspots’ in the helical domain (exon (Ex) 9) or the catalytic domain (Ex20) [2]. A somatic mutation in the plekstrin homology domain of *AKT1*:p.Glu17Lys is found in approximately 4% of breast tumors [3]. Conversely, recent evidence describing next generation sequencing showed that another key potential mechanism of the failure of ET involves activating mutations in the ligand-binding domain of the *ESR1* gene [4]. Those mutations cause ligand-independent estrogen receptor (ER) transcriptional activity that does not respond to endocrine manipulation. The estimated frequency from comprehensive studies was 20%-50% for *ESR1* mutations in MBC but that were minimally present in PBC. The potential of using *PI3K*/*AKT* pathway mutations and *ESR1* mutations as biomarkers to predict clinical response of ET is currently the focus of many preclinical and clinical studies such as the BOLERO-2 cohort [5]. Additionally, recent digital PCR assays on plasma cell-free DNA (cfDNA) of several cohorts demonstrated the difference in the clinical features between the representative hotspot mutations in MBC, *PIK3CA* and *ESR1* mutations [6–10]. In the BOLERO-2 study, Chandarlapaty and colleagues demonstrated that progression free survival (PFS) benefit of mammalian target of rapamycin (mTOR) inhibitor everolimus was maintained irrespective of *PIK3CA* mutations, but that was decreased according to the presence of *ESR1* mutations [6, 7]. In another two phase III randomized trials, Fribbens and colleagues reported the effectiveness of the target drug by having the mutations or not. In the SoFEA study, fulvestrant improved PFS of patients with *ESR1* mutations compared to exemestane. Meanwhile, in the PALOMA3 study, fulvestrant plus the CDK4/6-inhibitor palbociclib improved PFS regardless of the genomic status of *ESR1* or *PIK3CA* [8, 9]. We demonstrated the clinical significance of the burden of on-treatment hotspot *ESR1* mutations, both in a snapshot and serially in MBC patients in comparison with *PIK3CA* hotspot mutation status [10]. Although these studies revealed the clinical significance of the target mutations in the early treatment lines (TLs), the clinical significance of *PIK3CA*, *AKT1*, and *ESR1* mutations in late line treatment in ER+ BC patients is still controversial. In this retrospective study, we demonstrated the clinical significance of the burden of on-treatment hotspot mutations: *PIK3CA* Ex9:p.Glu542Lys/Val, Glu545Val/Gly/Ala/Gln/Lys and Gln546Leu/Arg/Pro/Glu/Lys and *PIK3CA* Ex20:p.His1047Leu/Arg/Tyr and Gly1049Arg/Ser and *AKT1*:p.Glu17Lys, and *ESR1:*p.Tyr537Ser/Asn and Asp538Gly in cfDNA in comparison with ER+ PBC patients in each TL of ER+ MBC patients using multiplex droplet digital PCR (ddPCR) assays.

## Results and discussion

**Fig. 1:**
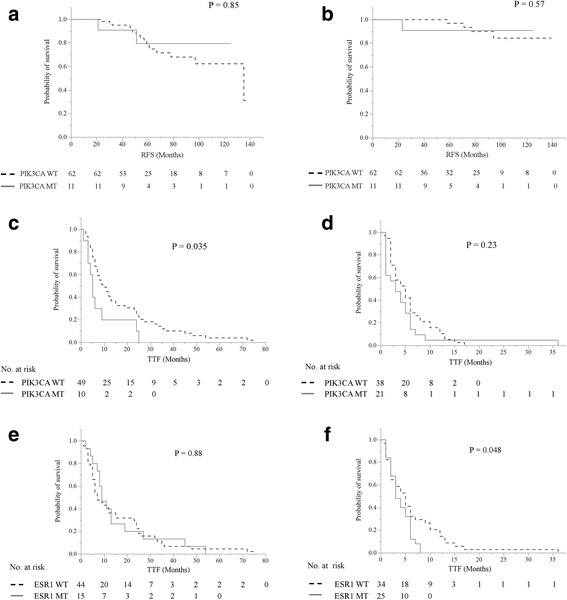
Kaplan-Meier plots of the association of the presence of cfDNA *PIK3CA* mutations with RFS; **a** and BCSS; **b** in the entire cohort and the presence of cfDNA *PIK3CA* mutations with TTF in less than the 5th line; **c** more than the 5th line; **d**, and the presence of cfDNA *ESR1* mutations with TTF in less than the 5th line; **e** and more than the 5th line; **f** in the entire cohort. The presence of cfDNA *PIK3CA* mutations and cfDNA *ESR1* mutations were defined as either positive or negative. Abbreviations: cfDNA, cell-free DNA; RFS, relapse-free survival; BCSS, breast cancer-specific survival; TTF, time to treatment failure

A total of 128 patients (251 plasma samples) with breast carcinoma who had an ECOG Performance scale status of 0 or 1 were enrolled in this study. The participants were comprised of 73 women (133 plasma samples) with PBC and 68 women (118 plasma samples) with MBC. Of these participants, 13 women had plasma samples with both PBC and MBC (Additional file 1: Table S1). Plasma *PIK3CA* mutations were found in 15.1% (11/73) of the PBC patients, the plasma *AKT1* mutation was detected in 1.4% (1/73), and the plasma *ESR1* mutations were observed in 2.7% (2/73) (Additional file 1: Table S2). One patient had co-mutations (*PIK3CA* mutations and *AKT1* mutation). This finding agrees with previous studies in which *PIK3CA* mutations are particularly common, whereas *AKT1* and *ESR1* mutations occur less frequently in PBC patients [3–5]. The presence of plasma *PIK3CA* mutations were statistically associated with invasive lobular carcinoma (*P* = 0.036) and marginally associated with histological grade III carcinoma (*P* = 0.059) (Additional file 1: Table S3). In the PBC group for recurrence free survival and breast cancer specific survival analyses, the patients were not stratified for the *PIK3CA* mutation status (Fig. 1a, b and Additional file 1: Tables S4, S5). In general, among the ER+ PBC patients, the *PIK3CA* mutations had varying clinical outcomes, but they were associated with a favorable prognosis [2]. Due to the difference in the clinical effect of *PIK3CA* mutations in the helical versus kinase domains, the prognostic effect may not be consistent. This finding suggests that the presence of Ex9 mutations predicted a more favorable outcome, whereas the presence of the Ex20 mutations predicted a relatively poor prognosis [2]. In this study, we could not evaluate the difference in the prognosis for each *PIK3CA* mutation, since *PIK3CA* Ex20 mutations were observed in 90.9% (10/11) of patients with *PIK3CA* mutations, and *PIK3CA* Ex9 and Ex20 mutations were observed in 10.1% (1/11) of patients with *PIK3CA* mutations (Additional file 1: Table S2). Interestingly, out of all of the patients, two patients with plasma *ESR1* mutations recurred. Investigations on the progression or survival by *ESR1* mutations in PBC have not been conducted and are highly desirable. Rudolph and colleagues reported that initial follow-up survival data suggests that the *AKT1*:p.Glu17Lys mutant could be associated with increased mortality [3]; however, none of the two patients with plasma *AKT1* mutation in this study showed recurrence.

**Fig. 2:**
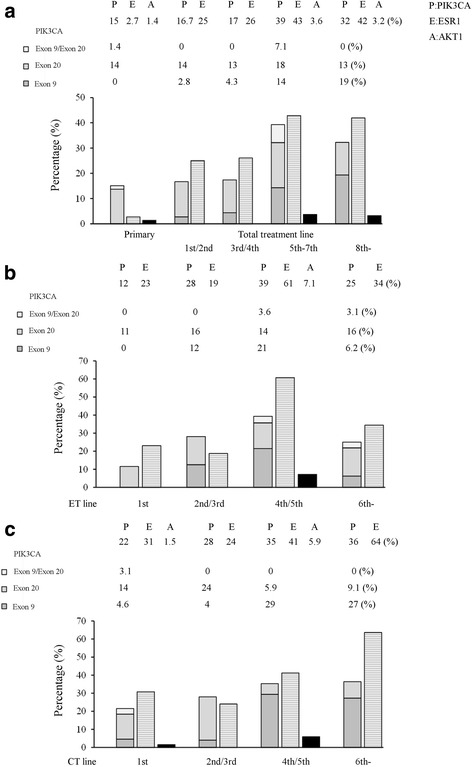
Distributions of *PIK3CA, AKT1,* and *ESR1* mutations according to each TL in ER-positive breast cancer. The bar graphs show *PIK3CA* mutations (gray monochrome)*, ESR1* mutations (gray horizontal stripes)*,* and *AKT1* mutation (black monochrome) from the left in each category. **a** The subgroup in the primary and each TL (the 1st/2nd line, the 3rd/4th line, the 5th–7th line, and the 8th line or more line) were omitted. The subgroup in each endocrine therapy line; **b**, and in each chemotherapy line; **c**, (the 1st line, the 2nd/3rd line, the 4th/5th line, and the 6th line or more line) were omitted. Abbreviations: ER, estrogen receptor; P, *PIK3CA* mutations; E, *ESR1* mutations; A, *AKT1* mutation

In the total TL analysis, as the TL increased, the frequency of *PIK3CA* and *ESR1* mutations increased due to the accumulation of genetic alterations. *PIK3CA* and *ESR1* mutations were specifically found to be lower in the 4th TL or less (*PIK3CA* vs *ESR1* mutations: 16.7% vs 25% in the 1st/2nd line and 17% vs 26% in the 3rd/4th line), compared with those in more than the 5th TL (*PIK3CA* vs *ESR1* mutations: 39% vs 43% in the 5th–7th line and 32% vs 42% in the 8th line or more) (Fig. 2a). In the ET line analysis of the MBC patients, *PIK3CA*, *AKT1*, and *ESR1* mutations were found to be highest in the 4th/5th line (*PIK3CA* mutations: 39% (11/28), *AKT1* mutation: 7.1% (2/28), and *ESR1* mutations: 61% (17/28)) (Fig. 2b). In the chemotherapy line analysis of the MBC patients, *PIK3CA* and *ESR1* mutations were found to be highest in the 6th line or more (*PIK3CA* mutations: 36% (4/11) and *ESR1* mutations: 64% (7/11)) (Fig. 2c). To the best of our knowledge, this result that *PIK3CA* and *ESR1* mutations were more frequent in the late TL is a new finding. Of these mutations, *ESR1* mutations were slightly more frequent than *PIK3CA* mutations. Meanwhile, plasma *AKT1* mutation was rare in MBC and it did not correlate with the TL (3.6% in the 5th–7th line and 3.2% in the 8th line or more) (Fig. 2). *AKT1* mutation coexisted with *PIK3CA* mutations in one MBC patient.

Next, we analyzed whether *PIK3CA* and *ESR1* mutations detected in cfDNA were associated with the differential benefit of time to treatment failure (TTF) of the early or late TL (Fig. 1c–f). By examining the difference in the TTF of the early or late TL with cfDNA *PIK3CA* and *ESR1* mutations, we could create subgroups of patients whose TL was either less than or more than the 5th line, since patients in less than the 5th line had a longer TTF than patients in more than the 5th line (*P* < 0.0001) (Additional file 1: Figure S1). Since the prevalence of the *AKT1* mutation is low in both PBC and MBC patients, the impact of *AKT1* mutation on prognosis remains unclear. Our data indicated that patients with cfDNA *PIK3CA* mutations had a shorter TTF than patients without mutations in the early TL group (*P* = 0.035), but there were no statistically significant differences in the patients with or without cfDNA *PIK3CA* mutations (*P* = 0.23) in the late TL group (Fig. 1c, d). Paradoxically, as the TL advanced, there was a tendency for *PIK3CA* Ex9 mutations, a more favorable mutation, to increase. *PIK3CA* Ex20 mutations, a more aggressive mutation, were expressed stably over the course of treatment (Fig. 2). Meanwhile, there was no statistically significant differences in patients with cfDNA *ESR1* mutations or not in the early TL group (*P* = 0.88), but patients with cfDNA *ESR1* mutations had a shorter TTF than patients without mutations in the late TL group (*P* = 0.048) (Fig. 1e, f). Additionally, as the TL advanced, there was a tendency for patients treated with ET to decrease (83% in the 1st/2nd line, 74% in the 3rd/4th line, 64% in the 5th–7th line, and 68% in the 8th line or more) (Additional file 1: Figure S2A) and there was a tendency for the best overall response for the treatment to be worse (the frequency of progressive disease: 33% in the 1st/2nd line, 30% in the 3rd/4th line, 50% in the 5th–7th line, and 45% in the 8th line or more) (Additional file 1: Figure S2B). These results suggested the presence of plasma *ESR1* mutations, which may be relevant for the choice of treatment of the ER+ MBC patients even in the higher TLs.

Recently, potential interest in the mutations accompanying disease progression or treatment has been increasing, since they may be a clue for the further treatment. Our data indicated that of the 65 PBC patients treated with neoadjuvant therapy, 9 patients (13.8%) acquired or maintained *PIK3CA* mutations, but 8 patients (12.3%) lost *PIK3CA* mutations after neo-adjuvant therapy. Meanwhile, one patient (1.5%) lost *AKT1* mutation and one patient (1.5%) acquired *ESR1* mutations after neo-adjuvant therapy. Of the 13 BC patients with both primary and recurrence blood samples, 6 patients (46.2%) acquired or maintained *PIK3CA* mutations, but 1 patient (7.7%) lost *PIK3CA* mutations during relapse. Meanwhile, 4 patients (30.8%) acquired or maintained *ESR1* mutations during relapse (Table 1). These results were very interesting, but we could not determine their clinical relevance due to the small sample size.

**Table 1:** Details of the change of *PIK3CA* mutations*, AKT1* mutation*,* and *ESR1* mutations

	No. of patients (%)														
	Changes of the mutations due to neo-adjuvant therapies (*N* = 65)								Changes of the mutations on relapse (*N* = 13)						
*PIK3CA*	E542x/ G1049x →H1047x	WT→ E542x /E545x, Q546x	WT→ H1047x	H1047x/ G1049x →WT	H1047x →WT	N/A→ H1047x	N/A →WT	WT →WT	H1047x→ H1047x	WT→ E542x/ E545x, Q546x /H1047x	WT→ E545x, Q546x	WT→ H1047x	WT→ G1049x	H1047x→WT	WT →WT
	1 (1.5)	2 (3.1)	6 (9.2)	1 (1.5)	7 (10.8)	1 (1.5)	4 (6.1)	43 (66.1)	1 (7.7)	1 (7.7)	1 (7.7)	2 (15.4)	1 (7.7)	1 (7.7)	6 (46.1)
*AKT1*	E17K→ WT	N/A→ WT	WT→ WT						WT→WT						
	1 (1.5)	5 (7.7)	59 (90.8)						13 (100)						
*ESR1*	N/A→ Y537S /Y537N /D538G	N/A→ WT	WT→ WT						Y537S/ Y537N /D538G→ Y537S /Y537N /D538G	WT→Y537S	WT→ D538G	WT→ WT			
	1 (1.5)	4 (6.1)	60 (92.3)						1 (7.7)	1 (7.7)	2 (15.4)	9 (69.2)			

*Abbreviations*: *WT* wild-type, *N/A* not available, *E542x* E542K/V, *E545x Q546x* E545V/G/A/Q/K Q546L/R/P/E/K, *H1047x* H1047L/R/Y, G1049x, G1049R/S

## Conclusions

Our study demonstrates the difference in the clinical significance of *PIK3CA*, *AKT1*, and *ESR1* hotspot mutations for each TL in ER+ BC patients using multiplex ddPCR assays. To the best of our knowledge, limited data exist on whether the detection of these mutations may be useful as a biomarker for predicting the effect of late line treatment.

## Additional file

Additional file 1: Methods and Supplementary information. (DOCX 407 kb)

## Abbreviations

cfDNA: cell-free DNA
ddPCR: droplet dPCR
ER: Estrogen receptor
ET: Endocrine therapy
Ex: Exon
MBC: Metastatic BC
PBC: Primary breast cancer
PFS: Progression free survival
TL: Treatment line
TTF: Time to treatment failure

## References

[1] Loi S, Haibe-Kains B, Majjaj S, Lallemand F, Durbecq V, Larsimont D, Gonzalez-Angulo AM, Pusztai L, Symmans WF, Bardelli A (2010). PIK3CA mutations associated with gene signature of low mTORC1 signaling and better outcomes in estrogen receptor-positive breast cancer. Proc Natl Acad Sci U S A.

[2] Dirican E, Akkiprik M, Ozer A (2016). Mutation distributions and clinical correlations of PIK3CA gene mutations in breast cancer. Tumour Biol.

[3] Rudolph M, Anzeneder T, Schulz A, Beckmann G, Byrne AT, Jeffers M, Pena C, Politz O, Kochert K, Vonk R, Reischl J (2016). AKT1 (E17K) mutation profiling in breast cancer: prevalence, concurrent oncogenic alterations, and blood-based detection. BMC Cancer.

[4] Angus L, Beije N, Jager A, Martens JW, Sleijfer S (2017). ESR1 mutations: moving towards guiding treatment decision-making in metastatic breast cancer patients. Cancer Treat Rev.

[5] Hortobagyi GN, Chen D, Piccart M, Rugo HS, Burris HA, 3rd, Pritchard KI, Campone M, Noguchi S, Perez AT, Deleu I, et al: Correlative analysis of genetic alterations and Everolimus benefit in hormone receptor-positive, human epidermal growth factor receptor 2-negative advanced breast cancer: results from BOLERO-2**.** J Clin Oncol 2016, 34**:**419–426. 10.1200/JCO.2014.60.1971 PMC5070556 26503204

[6] Moynahan ME, Chen D, He W, Sung P, Samoila A, You D, Bhatt T, Patel P, Ringeisen F, Hortobagyi GN (2017). Correlation between PIK3CA mutations in cell-free DNA and everolimus efficacy in HR+, HER2- advanced breast cancer: results from BOLERO-2. Br J Cancer.

[7] Chandarlapaty S, Chen D, He W, Sung P, Samoila A, You D, Bhatt T, Patel P, Voi M, Gnant M (2016). Prevalence of ESR1 mutations in cell-free DNA and outcomes in metastatic breast cancer: a secondary analysis of the BOLERO-2 clinical trial. JAMA Oncol.

[8] Cristofanilli M, Turner NC, Bondarenko I, Ro J, Im SA, Masuda N, Colleoni M, DeMichele A, Loi S, Verma S (2016). Fulvestrant plus palbociclib versus fulvestrant plus placebo for treatment of hormone-receptor-positive, HER2-negative metastatic breast cancer that progressed on previous endocrine therapy (PALOMA-3): final analysis of the multicentre, double-blind, phase 3 randomised controlled trial. Lancet Oncol.

[9] Fribbens C, O'Leary B, Kilburn L, Hrebien S, Garcia-Murillas I, Beaney M, Cristofanilli M, Andre F, Loi S, Loibl S (2016). Plasma ESR1 mutations and the treatment of estrogen receptor-positive advanced breast cancer. J Clin Oncol.

[10] Takeshita T, Yamamoto Y, Yamamoto-Ibusuki M, Tomiguchi M, Sueta A, Murakami K, Omoto Y, Iwase H (2017). Analysis of ESR1 and PIK3CA mutations in plasma cell-free DNA from ER-positive breast cancer patients. Oncotarget.

