# Supplementary material for: Clinical significance of plasma cell-free DNA mutations in PIK3CA, AKT1, and ESR1 gene according to treatment lines in ER-positive breast cancer
Source: Mol Cancer. 2018 Feb 26;17:67. doi: 10.1186/s12943-018-0808-y (PMC6389169; doi:10.1186/s12943-018-0808-y)

**Supplemental Methods and data**

**Methods**

**Patients and breast cancer samples**

A total of 128 patients (251 plasma samples) with breast carcinoma, treated at Kumamoto University Hospital between 2003 and 2017, were enrolled in this study. Cases were selected if archival plasma samples were available. Informed consent was obtained from all the patients before biopsy or surgery. The Ethics Committee of Kumamoto University Graduate School of Medicine (Kumamoto, Japan) approved the study protocol (#1857). We assessed and treated both PBC and MBC patients as described previously [1]. We defined the presence of progressive disease (PD) as a non- responder.

In the RFS analysis, local recurrences and distant metastases were considered to be an event. Seventeen (23.3 %) of the BC patients experienced relapse, and 56 (76.7 %) were relapse-free at the last follow-up. A total of 5 cases died from BC, which were regarded as events when analyzing the BCSS.

In the TTF analysis, discontinuation of ETs or CTs caused by local recurrences, distant metastases, and disease progression at any site following the blood draw were considered as an event.

**Sample preparation**

Blood collected in EDTA K_2_ tubes was processed as soon as possible and was centrifuged at 1,467 g for 10 min with plasma stored in a freezer until DNA extraction. DNA was extracted from 500 μL aliquots of plasma using the ISOSPIN Blood & Plasma DNA kit (Nippon Gene, Tokyo, Japan) according to the manufacturer’s instructions. All DNA extracts were quantified using a NanoDrop 2000 spectrometer (NanoDrop Technologies, Wilmington, DE, USA), and purity was determined from the A260/A280 absorbance ratios.

**Analysis of *PIK3CA*, *AKT1*, and *ESR1* mutations by ddPCR**

We performed duplicate ddPCR assays on a QX200 digital PCR system (Bio-Rad laboratories, Hercules, CA, USA) using the assays as described previously [1]. The PCR data were quantified as copies/μL using QuantaSoft^™^ software (Bio-Rad laboratories). A mutation was considered positive with more than three *PIK3CA, AKT1,* and *ESR1* mutant droplets. The uniplex ddPCR method had been optimized beforehand by comparative analysis of a dilution series of synthetic copies of each indicated mutant oligonucleotide, as reported previously [1].

**Probes and primers**

We used LBx® Probe PIK3CA Screen1 (A087) as the detection probe for *PIK3CA* E542K/V and E545V/G/A/Q/K, Q546L/R/P/E/K, and LBx® Probe PIK3CA Screen2 (A088) as the detection probe for *PIK3CA* H1047L/R/Y and G1049R/S (Riken Genesis, Tokyo, Japan). Additionally, we used LBx® Probe AKT1 E17K (A084) as the detection probe for *AKT1* E17K, and LBx® Probe ESR1 Multi (A082) as the detection probe for *ESR1* Y537S/Y537N, and D538G.

**Immunohistochemistry**

Immunohistochemical staining was carried out on 4-μm-thick tumor sections. Serial sections were prepared from the selected blocks and float-mounted on the adhesive-coated glass slides for the ERα, progesterone receptor (PgR), human epidermal growth factor receptor 2 (HER2), and Ki67 staining. Primary antibodies, their visualization methods, and their evaluation were as previously described [2].

**Statistical analysis**

The chi-square test or Fisher’s exact test were used to assess the baseline differences between the binary variables. Correlations were calculated using Spearman’s rank correlation coefficient. In the analysis of recurrence free survival (RFS), breast cancer specific survival (BCSS), and time to treatment failure (TTF). The Kaplan–Meier method was used to estimate survival rates, and differences between the survival curves were evaluated by the log-rank test. Cox’s proportional hazards model was used for the univariate and multivariate analysis of the prognostic status. *P* values < 0.05 were considered to be a significant result. All of the reported *P* values are two-sided, and confidence intervals (CIs) are at the 95 % level. All the statistical analyses were two-sided and performed using JMP software version 10.0.1 for Windows (SAS Institute Japan, Tokyo, Japan).

**Reference**

1. Takeshita T, Yamamoto Y, Yamamoto-Ibusuki M, Tomiguchi M, Sueta A, Murakami K, Omoto Y, Iwase H: **Analysis of ESR1 and PIK3CA mutations in plasma cell-free DNA from ER-positive breast cancer patients.** *Oncotarget* 2017, **8:**52142-52155.

2. Takeshita T, Omoto Y, Yamamoto-Ibusuki M, Yamamoto Y, Iwase H: **Clinical significance of androgen receptor and its phosphorylated form in breast cancer.** *Endocr Relat Cancer* 2013, **20:**L15-21.

**Tables**

Table S1. MBC Patients’ characteristics

|  | No. of patients (%) |
| --- | --- |
| **Variables** | (*N* = 68) |
| **Age at the first blood draw** |  |
| Median (range) | 58 (50–66) |
| **Histological type** |  |
| Invasive ductal | 65 (95.6) |
| Invasive lobular | 1 (1.5) |
| Mucinous | 1 (1.5) |
| Neuroendocrine | 1 (1.5) |
| **Histological grade** |  |
| 1 | 18 (26.5) |
| 2 | 28 (41.2) |
| 3 | 16 (23.5) |
| Lobular | 1 (14.7) |
| Unknown | 5 (7.4) |
| **The percentage of ERα median (25%, 75%)** | 90 (70–95) |
| **PgR** |  |
| Negative | 21 (30.9) |
| Positive | 46 (67.6) |
| Unknown | 1 (14.7) |
| **HER2** |  |
| Negative | 58 (85.3) |
| Positive | 10 (14.7) |
| **Visceral involvement** |  |
| No | 18 (26.5) |
| Yes | 50 (73.5) |
| **Bone involvement** |  |
| No | 29 (42.6) |
| Yes | 39 (57.4) |
| **The number of metastasis lesion** |  |
| 1 | 8 (11.8) |
| 2 | 23 (33.8) |
| 3 ≤ | 37 (54.4) |
| **The treatment line at the first blood draw** |  |
| 1st | 24 (35.3) |
| 2nd | 8 (11.8) |
| 3rd | 9 (13.2) |
| 4th | 3 (4.4) |
| 5th | 5 (7.3) |
| 6th | 3 (4.4) |
| More than 7th | 16 (23.5) |
| **The number of the blood draw** |  |
| 1 | 18 (26.5) |
| 2 | 35 (51.5) |
| 3 | 12 (17.6) |
| 4 | 2 (2.9) |
| 5 | 1 (1.5) |
| **Possession of the blood specimen of PBC** | 13 (19.1) |
| **Outcome** |  |
| Death | 33 (48.5) |

Abbreviations: MBC, metastatic breast cancer; ERα, estrogen receptor alpha**;** PgR, progesterone receptor; HER2, human epidermal growth factor receptor 2; PBC, primary breast cancer.

Table S2. PBC patients’ characteristics

|  | No. of Patients (%) |
| --- | --- |
| **Variables** | (*N* = 73 ) |
| **Age at diagnosis** |  |
| Median (range) | 56 (31-82) |
| **Clinical Stage at diagnosis** |  |
| I | 10 (13.7) |
| II | 46 (63.0) |
| III | 17 (23.3) |
| **Histological type** |  |
| Invasive ductal | 69 (94.5) |
| Invasive lobular | 1 (1.4) |
| Mucinous | 3 (4.1) |
| **Histological grade** |  |
| 1 | 22 (30.1) |
| 2 | 35 (47.9) |
| 3 | 12 (16.4) |
| Lobular | 1 (1.4) |
| Mucinous | 3 (4.1) |
| **Percentage of ERα median (25%, 75%)** | 90 (70–93.7) |
| **Percentage of PgR median (25%, 75%)** | 45 (5–77.5) |
| **HER2** |  |
| Negative | 64 (87.7) |
| Positive | 9 (12.3) |
| **Ki67** |  |
| Negative | 30 (41.1) |
| Positive | 26 (35.6) |
| Unknown | 17 (23.3) |
| **Somatic mutations** |  |
| *PIK3CA* total mutations | 11 (15.1) |
| ^#^*PIK3CA* exon 9 mutations | 0 |
| ^$^*PIK3CA* exon 20 mutations | 10 (90.9) |
| *PIK3CA* exon 9 and 20 mutations | 1 (10.1) |
| *AKT1* E17K | 1 (1.4) |
| ^&^*ESR1* mutations | 2 (2.7) |
| **Primary therapy** |  |
| NAC | 46 (63) |
| NET | 19 (26) |
| Operation | 8 (11) |

Abbreviations: PBC, primary breast cancer; ERα, estrogen receptor alpha; PgR, progesterone receptor; HER2, human epidermal growth factor receptor 2; NAC, neoadjuvant chemotherapy, NET, neoadjuvant endocrine therapy.

^#^*PIK3CA* exon 9 mutations were *PIK3CA* E542K/V and E545V/G/A/Q/K, Q546L/R/P/E/K.

^$^*PIK3CA* exon 20 mutations were *PIK3CA* H1047L/R/Y and G1049R/S

^&^*ESR1* mutations were *ESR1* Y537S/Y537N, and D538G.

Table S3. Patient characteristics associated with *PIK3CA, AKT1,* and *ESR1* status in PBC.

|  | No. of patients (%) | | | | | | | | |
| --- | --- | --- | --- | --- | --- | --- | --- | --- | --- |
| **Variables** | Total | *PIK3CA* | | | *AKT1* | | *ESR1* | | |
|  |  | Wild-type | Mutant^”^ | *P*-value | Wild-type | Mutant^$^ | Wild-type | Mutant^&^ | *P*-value |
|  | (*N* = 73) | (*N* = 62 ) | (*N* = 11 ) |  | (*N* = 72) | (*N* = 1) | (*N* = 71) | (*N* = 2) |  |
| **Age at biopsy** |  |  |  |  |  |  |  |  |  |
| ≤ 50 | 29 (38.9) | 24 (38.7) | 5 (45.5) | 0.67 | 29 (40.3) | 0 | 28 (39.4) | 1 (50) | 0.76 |
| > 50 | 44 (61.1) | 38 (61.3) | 6 (54.5) |  | 43 (59.7) | 1 | 43 (60.6) | 1 (50) |  |
| **Clinical Stage at diagnosis** |  |  |  |  |  |  |  |  |  |
| I, II | 56 (76.7) | 46 (74.2) | 10 (90.9) | 0.22 | 55 (76.4) | 1 | 54 (76.1) | 2 (100) | 0.43 |
| III | 17 (23.3) | 16 (25.8) | 1 (9.1) |  | 17 (23.6) | 0 | 17 (23.9) | 0 |  |
| **Histological type** |  |  |  |  |  |  |  |  |  |
| Invasive ductal | 69 (94.5) | 60 (96.8) | 9 (81.8) | 0.036^a^ | 68 (94.4) | 1 | 67 (94.4) | 2 (100) | 0.94 |
| Invasive lobular | 1 (1.4) | 0 | 1 (9.1) |  | 1 (1.4) | 0 | 1 (1.4) | 0 |  |
| Mucinous | 3 (4.1) | 2 (3.2) | 1 (9.1) |  | 3 (4.2) | 0 | 3 (4.2) | 0 |  |
| **Histological grade** |  |  |  |  |  |  |  |  |  |
| 1, 2 | 57 (78.1) | 51 (82.3) | 6 (54.5) | 0.059 | 57 (79.1) | 0 | 56 (78.9) | 1 (50) | 0.42 |
| 3 | 12 (16.4) | 9 (14.5) | 3 (27.3) |  | 11 (15.3) | 1 | 11 (15.5) | 1 (50) |  |
| Lobular/ Mucinous | 4 (5.5) | 2 (3.2) | 2 (18.2) |  | 4 (5.6) | 0 | 4 (5.6) | 0 |  |
| **Percentage of ERα median (25%, 75%)** | 90  (50–93.7) | 90  (70–92.5) | 90  (80–95) | 0.74 | 90  (70-95) | 80 | 90  (70-95) | 80  (70-90) | 0.64 |
| **PgR** |  |  |  |  |  |  |  |  |  |
| Negative | 11 (15.2) | 8 (12.9 | 3 (27.3) | 0.22 | 11 (15.3) | 0 | 10 (14.1) | 1 (50) | 0.16 |
| Positive | 62 (84.7) | 54 (87.1) | 8 (72.7) |  | 61 (84.7) | 1 | 61 (85.9) | 1 (50) |  |
| **HER2 positivity** |  |  |  |  |  |  |  |  |  |
| Negative | 64 (87.7) | 55 (88.7) | 9 (81.8) | 0.54 | 63 (87.5) | 1 | 62 (87.3) | 2 (100) | 0.59 |
| Positive | 9 (12.3) | 7 (11.3) | 2 (18.2) |  | 9 (12.5) | 0 | 9 (12.7) | 0 |  |
| **Ki67 LI** |  |  |  |  |  |  |  |  |  |
| Negative | 30 (41.1) | 24 (38.7) | 6 (54.5) | 0.39 | 30 (41.7) | 0 | 29 (40.8) | 1 (50) | 0.92 |
| Positive | 26 (35.6) | 23 (37.1) | 3 (27.3) |  | 26 (36.1) | 0 | 25 (35.2) | 1 (50) |  |
| Unknown | 17 (23.2) | 15 (24.2) | 2 (18.2) |  | 16 (22.2) | 1 | 17 (23.9) | 0 |  |
| **Primary therapy** |  |  |  |  |  |  |  |  |  |
| NAC | 46 (63) | 40 (64.5) | 6 (54.5) | 0.70 | 45 (62.5) | 1 | 46 (64.8) | 0 | 0.1 |
| NET | 19 (26) | 15 (24.2) | 4 (36.4) |  | 19 (26.4) | 0 | 18 (25.4) | 1 (50) |  |
| Operation | 8 (11) | 7 (11.3) | 1 (9.1) |  | 8 (11.1) | 0 | 7 (9.9) | 1 (50) |  |
| **Outcome** |  |  |  |  |  |  |  |  |  |
| Recurrence | 17 (22.2) | 15 (24.2) | 2 (18.2) | 0.66 | 17 (23.6) | 0 | 15 (21.1) | 2 (100) | 0.0093^a^ |
| Death | 5 (6.8) | 4 (6.5) | 1 (9.1) | 0.75 | 5 (6.9) | 0 | 5 (7) | 0 | 0.70 |

^#^*PIK3CA* mutations were *PIK3CA* E542K/V, E545V/G/A/Q/K and Q546L/R/P/E/K, H1047 L/R/Y, and G1049R/S.

^#^*AKT* mutation was *AKT1* E17K

^&^*ESR1* mutations were *ESR1* Y537S/Y537N and D538G

Abbreviations: PBC, primary breast cancer; ERα, estrogen receptor alpha; PgR, progesterone receptor; HER2, human epidermal growth factor receptor 2; LI, labeling index; NAC, neoadjuvant chemotherapy; NET, neoadjuvant endocrine therapy.

^a^ Factor showing statistical significance.

Table S4. Univariate and multivariate analysis of factors associated with progression free survival in women with primary breast cancer. (Cox proportional hazards model)

| **Variables** |  |  | Univariate analysis | | | Multivariate analysis | | |
| --- | --- | --- | --- | --- | --- | --- | --- | --- |
|  |  | Value | HR | 95%CI | ***P*** value | HR | 95%CI | ***P*** value |
| **Age at biopsy** | (ref = ≤ 50) | > 50 | 1.22 | 0.45-3.62 | 0.70 |  |  |  |
| **Clinical Stage at diagnosis** | (ref = I,II) | III | 1.37 | 0.43-3.74 | 0.57 | 1.41 | 0.44-4.03 | 0.54 |
| **Histological grade** | (ref = 1,2) | 3 | 0.67 | 0.11-2.43 | 0.58 | 0.67 | 0.10-2.47 | 0.59 |
| **ER (IHC)** | (ref = median) | ≥ 90% | 0.41 | 0.065-1.50 | 0.20 |  |  |  |
| **PgR (IHC)** | (ref ≤ 1%) | > 1% | 0.44 | 0.15-1.59 | 0.19 |  |  |  |
| **HER2 positivity** | (ref = Negative) | Positive | 1.21 | 0.19-4.34 | 0.81 |  |  |  |
| **Ki67 LI** | (ref = ≤ 14) | > 14 | 0.84 | 0.24-2.79 | 0.77 |  |  |  |
| **Primary therapy** | (ref = NET) | NAC | 1.02 | 0.24-6.92 | 0.97 |  |  |  |
| ***PIK3CA* genomic state** | (ref = WT) | Total MT | 0.87 | 0.13-3.11 | 0.85 | 1.12 | 0.17-4.31 | 0.89 |
|  | (ref = WT) | E9 MT | 1,4751e-9 | 9.93 | 0.52 |  |  |  |
|  | (ref = WT) | E20 MT | 0.86 | 0.13-3,11 | 0.85 |  |  |  |

Abbreviations: HR, hazard ratio; 95%CI, 95% confidence interval; ER, estrogen receptor; IHC, immunohistochemistry; PgR, progesterone receptor; HER2, human epidermal growth factor receptor 2; LI, labeling index; NET, neoadjuvant endocrine therapy; NAC, neoadjuvant chemotherapy; WT, wild-type; MT, mutation, E9, exon 9; E20, exon 20.

^#^E9 MT was *PIK3CA* E542K/V, E545V/G/A/Q/K, and Q546L/R/P/E/K.

^$^E20 MT was *PIK3CA* H1047L/R/Y and G1049R/S

Table S5. Univariate and multivariate analysis of factors associated with breast cancer specific survival in women with primary breast cancer. (Cox proportional hazards model)

| **Variables** |  |  | Univariate analysis | | | Multivariate analysis | | |
| --- | --- | --- | --- | --- | --- | --- | --- | --- |
|  |  | Value | HR | 95%CI | ***P*** value | HR | 95%CI | ***P*** value |
| **Age at biopsy** | (ref = ≤ 50) | > 50 | 1.37 | 0.23-10.6 | 0.73 |  |  |  |
| **Clinical Stage at diagnosis** | (ref = I,II) | III | 1.3053e-9 | 1.47-1.47 | 0.09 | 1.2136e-9 | 1.967e-35-1,52 | 0.099 |
| **Histological grade** | (ref = 1,2) | 3 | 4.0891e-9 | 0-0.302 | 0.23 | 1.3718e-9 | 0-2.91 | 0.22 |
| **ER (IHC)** | (ref = median) | ≥ 90% | 2.45 | 0.32-14.9 | 0.35 |  |  |  |
| **PgR (IHC)** | (ref ≤ 1%) | > 1% | 0.54 | 0.077-10.6 | 0.60 |  |  |  |
| **HER2 positivity** | (ref = Negative) | Positive | 2.20 | 0.11-15.0 | 0.52 |  |  |  |
| **Ki67 LI** | (ref = ≤ 14) | > 14 | 3.54 | 0.45-71.7 | 0.24 |  |  |  |
| **Primary therapy** | (ref = NET) | NAC | 143785515 | 0.16- | 0.33 |  |  |  |
| ***PIK3CA* genomic state** | (ref = WT) | MT | 1.86 | 0.094-12.9 | 0.60 | 1.32 | 0.067-9.04 | 0.81 |
|  | (ref = WT) | E9 MT^#^ | 2.9929e-7 | 419 | 0.87 |  |  |  |
|  | (ref = WT) | E20 MT^$^ | 1.86 | 0.094-12.9 | 0.60 |  |  |  |

Abbreviations: HR, hazard ratio; 95%CI, 95% confidence interval; ER, estrogen receptor; IHC, immunohistochemistry; PgR, progesterone receptor; HER2, human epidermal growth factor receptor 2; LI, labeling index; NET, neoadjuvant endocrine therapy; NAC, neoadjuvant chemotherapy; WT, wild-type; MT, mutation, E9, exon 9; E20, exon 20.

^#^E9 MT was *PIK3CA* E542K/V, E545V/G/A/Q/K, and Q546L/R/P/E/K.

^$^E20 MT was *PIK3CA* H1047L/R/Y and G1049R/S

**Figures**

**Fig. S1.** Kaplan-Meier plots of the association of each treatment line groups with the patient response end-points or TTF in the entire cohort. The subgroup in the 1^st^/2^nd^ treatment line, the 3^rd^/4^th^ treatment line, the 5^th^-7^th^ treatment line, and the 8^th^ treatment line or more were omitted

Abbreviations: TTF, time to treatment failure.


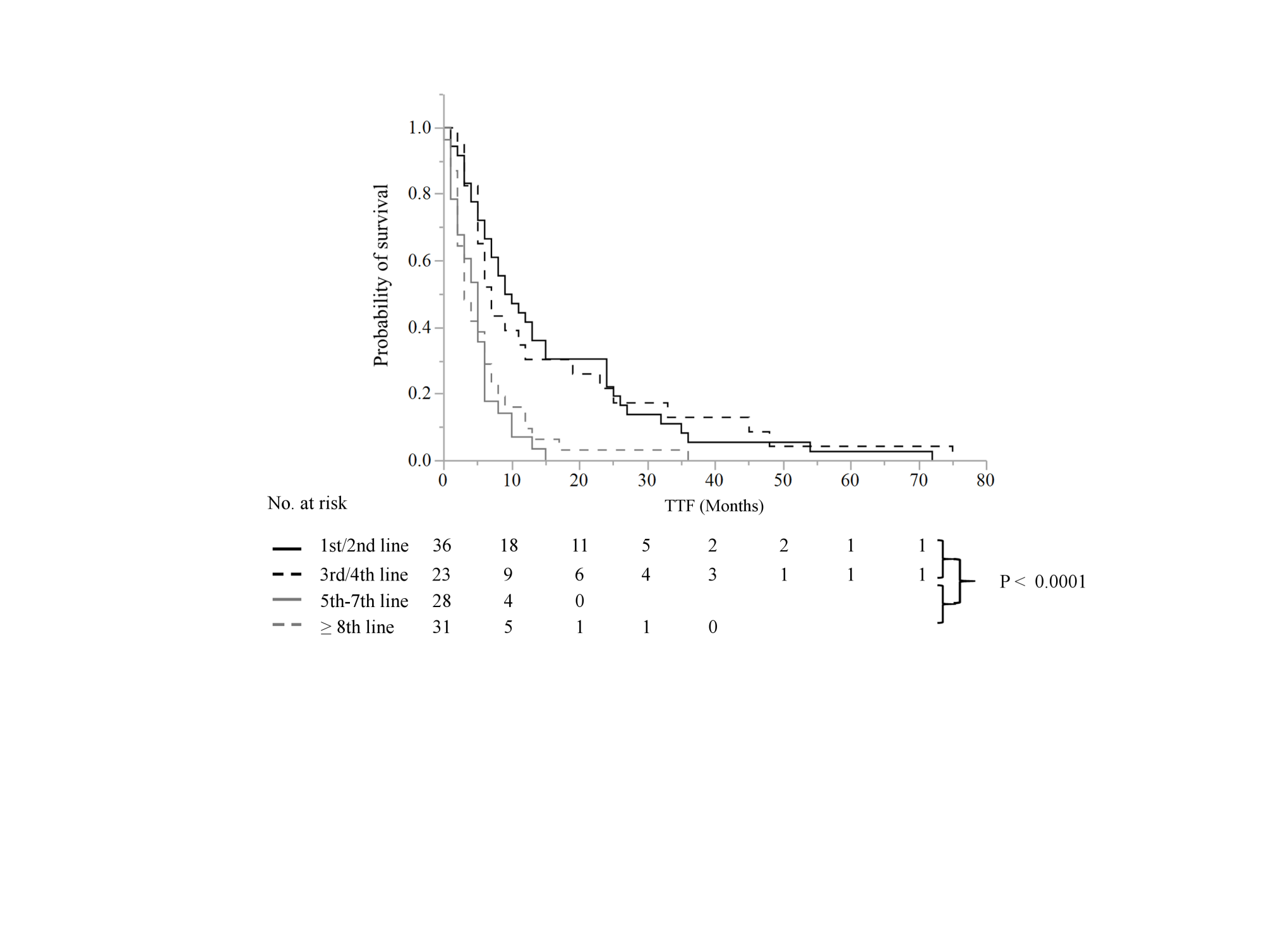


**Fig. S2.** Distributions of the type of treatment; **A**, and BOR for therapy; **B**, according to each treatment line in comparison with *PIK3CA* and *ESR1* mutations in ER-positive breast cancer. Type of treatment was classified as ET or CT; **A**, and BOR was classified as PD or non-PD, **B**. The subgroup in the 1^st^/2^nd^ line, the 3^rd^/4^th^ line, the 5^th^-7^th^ line, and the 8^th^ line or more were omitted. *PIK3CA* mutation was indicated by a solid line and *ESR1* mutation was indicated by a broken line.

Abbreviations: BOR, best overall response; ER, estrogen receptor, ET, endocrine therapy; CT, chemotherapy; MT, mutation; PD, progressive disease.


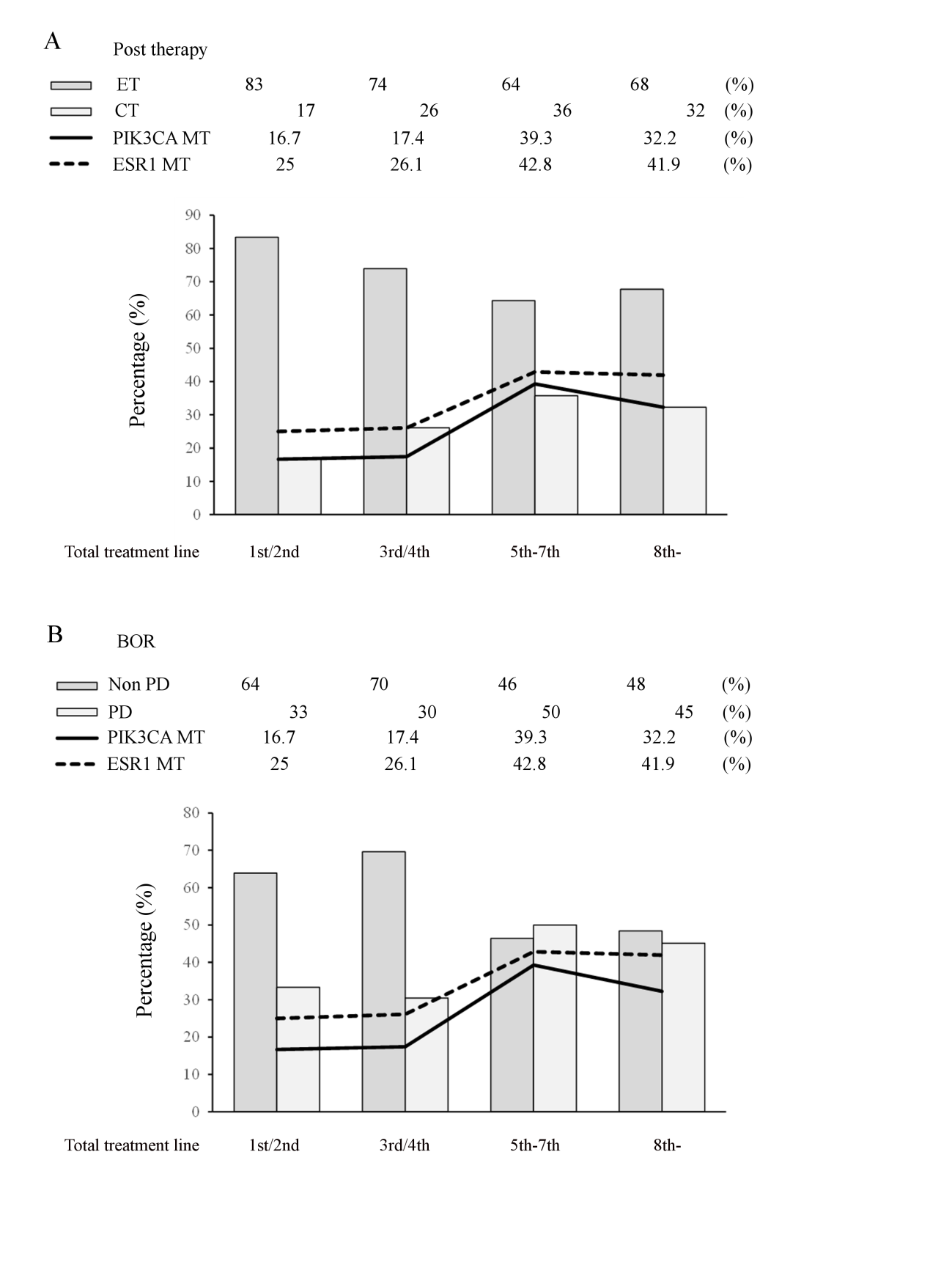

Supplement: Supplementary file 1 — Methods and Supplementary information. (DOCX 407 kb) [file 12943_2018_808_MOESM_ESM.docx]
